# Children’s Influence on Their Parents’ Satisfaction with Physical Activity in Nature: An Exploratory Study

**DOI:** 10.3390/ijerph20065093

**Published:** 2023-03-14

**Authors:** Jorge Rojo-Ramos, Antonio Castillo-Paredes, María Mendoza-Muñoz, José Carmelo Adsuar, Irene Polo-Campos, Santiago Gomez-Paniagua, Carmen Galán-Arroyo

**Affiliations:** 1 Physical Activity for Education, Performance and Health, Faculty of Sport Sciences, University of Extremadura, 10003 Cáceres, Spain; 2 Grupo AFySE, Investigación en Actividad Física y Salud Escolar, Escuela de Pedagogía en Educación Física, Facultad de Educación, Universidad de Las Américas, República 71, Santiago 8370040, Chile; 3 Research Group on Physical and Health Literacy and Health-Related Quality of Life (PHYQOL), Faculty of Sport Sciences, University of Extremadura, 10003 Cáceres, Spain; 4 Promoting a Healthy Society Research Group (PHeSO), Faculty of Sport Sciences, University of Extremadura, 10003 Cáceres, Spain; 5 BioẼrgon Research Group, Faculty of Sport Sciences, University of Extremadura, 10003 Cáceres, Spain

**Keywords:** physical activity in nature, satisfaction, parents, children, gender, age

## Abstract

Physical activity in nature has several benefits as it is important for good health, offering physical, social, psychological or even ecological benefits. Nevertheless, in order to maintain adherence to this practice, high levels of satisfaction with the practice are necessary. The objective of this study is to explore whether children’s characteristics influence parental satisfaction with physical activity in nature, analyzing possible differences according to the gender and age of their children. Two hundred and eighty parents responded to two sociodemographic questions in addition to the Physical Activity Enjoyment Scale (PACES), which consists of 16 items. The normality of the data was determined using the Kolmogorov-Smirnov test. Subsequently, nonparametric tests were used to analyze the variables of gender and age in the items, dimensions and total scores of the questionnaire. Statistical differences were found in some of the positive items, which varied according to the age of the children. However, no significant differences were found in the items with respect to the children’s gender or when examining the dimensions or total score of the questionnaire based on both variables. Likewise, age did not show significant correlations with the dimensions and the total score of the questionnaire. Consequently, this study indicates that a child’s age may influence parents’ positive perceptions of the enjoyment of physical activity in nature. Similarly, the gender of the child does not seem to influence these perceptions.

## 1. Introduction

Sedentary habits and low levels of physical activity (PA) have been shown to have negative effects on human health [1]. According to the World Health Organization (WHO), “health” is a condition of whole physical, mental, and social well-being and not merely the absence of sickness or disability [2]. The WHO lists pediatric physical inactivity as one of its top priorities. Over the past 20 years, physical inactivity has become widespread [3]. In this context, different studies showed that increased sedentary lifestyles, linked to chronic diseases such as childhood obesity, may, for the first time, put today’s children at risk of living shorter lives than their parents [4]. Regular PA in outdoor settings has been suggested as a useful method for contrasting noncommunicable diseases and chronic pathologies all over the world [5]. Outdoor play has been more popular recently as a way to boost PA and fend off risk factors like obesity, hypertension, and dyslipidemia [6].

Exercise in a natural setting, as opposed to indoor or outdoor built surroundings, improves concentration, decreases negative emotions, and increases energy and a sense of renewal; participants have a greater desire to return to the activity in the future and express greater happiness and satisfaction with outdoor exercise [7]. Since this type of activity is linked with enjoyment, curiosity, effort, the desire to participate and the intention to continue practicing an activity, it has positive effects on young people [8]. This activity also leads to a more self-determined or intrinsic motivation [9]. In both the short (immediately after exposure) [10] and long-term (during the following four weeks) [11], green exercise has been linked to improved mental health. Another finding that corroborated the aforementioned data was the beneficial relationship between overall health and PA in nature [12]. In this context, an increasing number of studies demonstrate the importance of public and green areas planned for PA in cities [13] because they can reduce social problems and offer ecological, physical, psychological, social, health, and economic benefits [14,15]. Moreover, research by White et al. [16] showed a weak but substantial correlation between life satisfaction and proportions of green space in towns of no more than 1500 inhabitants.

Motivation may also be a significant component. People frequently engage in leisure-time PA because they find it enjoyable or believe it will benefit them [17]; therefore, the key to autonomous motivation is making the decision to participate for enjoyment [18]. Following the self-determination theory, actions made out of autonomous motivation are more likely to be connected to the satisfaction of psychological needs (such as autonomy, competence, and relatedness), and when those needs are met, well-being is increased [19]. Consequently, the higher the level of enjoyment a person achieves with a given activity, the greater will be his or her satisfaction and, therefore, his or her adherence to it. By understanding this mechanism, it is anticipated that actions conducted out of autonomous motivation will result in more favorable psychological outcomes than actions taken out of controlled motivation [20]. In this sense, a person’s level of satisfaction can be understood as the subjective cognitive evaluation of their life and all of its facets, taking into account their standards of living, expectations and aspirations, and the goals they have attained; this evaluation is based on their own standards and is made in a positive way [21]. Additionally, research demonstrates that exercise with a choice is more likely to boost pleasant emotions and satisfaction than exercise without an option [22].

Conversely, the transition to parenthood is challenging since it requires learning new habits and parenting skills as well as dealing with worry about becoming a parent [23]. In addition, the responsibilities of any professional or domestic roles must be modified, and there are effects on mental and physical well-being [24]. Parents of small children have the risk of becoming sedentary [25], as the activities performed before parenthood often change afterward; therefore, leisure activities are often replaced by domestic and childcare activities [26]. Moreover, parents often experience feelings of guilt related to spending time away from their children for exercise, given that the time they have with their children is already restricted, further worsening people’s perception of how little time they have [27]. Research reports that 55.2% of women quit working out before becoming pregnant [28], even though the negative effects of inactivity could be a serious issue for pregnant women [29]. For example, pregnancy-related health advantages of exercise include a reduction in gestational diabetes and gestational hypertension [30]. The fact that infants of physically active mothers have been reported to be more interested in exploring their surroundings is one of the observed fetal health benefits of maternal exercise [31]. For people who are already physically active and belong to more disadvantaged groups, living in green residential areas has been shown to reduce depressive symptoms during pregnancy [32]. However, work-life balance issues have a negative impact on people’s health, with working mothers with young children facing significant problems [33]. Reducing the amount of time spent sleeping or exercising can frequently help people overcome their difficulty balancing work and family life [34]. On the other hand, studies have shown that the more physical exercise parents do during their free time, the more their children do as well, thus demonstrating similarities in the PA behavior of adults and children [35]. In addition, parental warmth has been found to be positively associated with child PA [36]. In this context, the literature indicates that there is a new trend in terms of lack of time for PA, affecting both parents equally, in contrast to early research that indicated that mothers were the ones who were primarily affected [37].

Generally, the literature is predominated by studies that focus on how parents are influencing their children rather than how children are influencing their parents [38]. So, once we know in general terms the importance of PA in the natural environment, its multiple benefits in the short and long term, and the need to continue doing PA before and after becoming parents, the objective of this study is to find out if the satisfaction experienced by parents when doing PA in nature is influenced by the characteristics of their children.

## 2. Materials and Methods

### 2.1. Participants

Participants were chosen using a non-probabilistic sample technique based on convenience sampling [39]. The study included 280 parents in total. There were 63.9% women and 36.1% men overall.

### 2.2. Instruments and Measures

The questionnaire that was administered to parents through the Google Forms tool obtained the sociodemographic data of the participants. In order to assess the degree of enjoyment with physical exercise, the Spanish version of the Physical Activity Enjoyment Scale (PACES) [40] questionnaire was also used. Each of the 16 components that make up this instrument is preceded by the phrase “when I am active in nature (doing physical activity, physical exercise, or playing a sport…)”. A Likert-type scale is used, with values ranging from 1 to 5, where 1 is used to indicate “complete disagreement” and 5 is used to indicate “full agreement.” Nine of the sixteen questions are about favorably accepting PA, while seven are about adversely rejecting PA. Since the scale’s application yields a score based on the sum of all the things, with 16 serving as the minimum value for a low level of enjoyment of PA and 80 acting as the highest value for that activity, the negative elements were reversed. The authors of the original study obtained Cronbach’s Alpha reliability values of 0.89 for the scale adapted to the Spanish language [40].

### 2.3. Procedures

The Google Forms software was used to design the sociodemographic and PACES data surveys. It made it simpler to reduce expenses, distribute questionnaires to participants, and keep their responses in a single database [41]. Between September and December of 2022, the data were collected.

The database of public schools in the Autonomous Community of Extremadura (Spain) maintained by the Department of Education and Employment of the Regional Government of Extremadura was utilized to access the sample (available at: http://estadisticaeducativa.educarex.es/?centros/ensenanzas/&curso=17&ensenanza_centro=101200001, accessed on 30 September 2022). The centers providing the second stage of early childhood education were picked, and contact information was chosen (3 to 6 years). The early childhood education teachers were then emailed with study information and requested to participate. The schools interested in participating were given the informed consent form, which required the participants’ (parents’) signatures. The PACES questionnaire, which comprised the sociodemographic questions, was sent to the parents in electronic form by a URL once they had consented to participate and signed the informed consent form.

The decision was made to phone the center and send an email again informing them of the study and how to participate in it after the first month’s response rate was deemed insufficient. The sample was gradually expanded in order to collect the necessary data.

### 2.4. Statistical Analysis

The information gathered from the surveys was examined using the Statistical Package for Social Sciences (SPSS) version 23.0 for MAC. The Kolmogorov-Smirnov test was used to determine whether the data were normally distributed. Nonparametric tests were utilized since the results of this test showed that the assumption was false. The median (Me) and interquartile range (IQR) are used to present descriptive data.

To analyze the possible significant differences in the items of the questionnaire, their dimensions and the total scale, according to the sociodemographic variables of the children, the Mann-Whitney U test was used to explore gender and the Kruskal-Wallis test to evaluate the three different ages. In addition, Spearman’s Rho correlation test was used to check the relationship between each dimension and the age of the children. Finally, the reliability of the instrument was evaluated using Cronbach’s Alpha. Bernstein [42] claims that dependability levels between 0.60 and 0.70 are acceptable, whereas values between 0.70 and 0.90 are satisfactory. The level of statistical significance was set at *p* < 0.05.

## 3. Results

The distribution of frequencies according to the gender and age of the parents can be seen in Table 1.

Table 2 provides information about the characteristics of the children. It takes into account three variables: gender, the grade of the preschool course in which they are enrolled and age.

Table 3 presents descriptive data based on the median (Me) and interquartile range (IQR) for each gender and age for each question on the PACES questionnaire. The Mann-Whitney U test was used to analyze differences between gender, and the Kruskal-Wallis test was used for differences in ages.

Overall, no significant differences were found in terms of gender and age of the children in the influence they have on their parents’ satisfaction with PA in nature. However, in four positive items (6, 10, 14 and 15), significant differences were found for the age of their children (*p* = 0.003, *p* = 0.000, *p* = 0.001 and *p* = 0.047).

According to gender and age, Table 4 displays the total score and median Likert score obtained on the PACES.

Regarding the dimensions and total scores of the questionnaire, no significant differences were observed in the responses when compared to the two grouping variables.

The Spearman’s Rho test was used to examine the relationship between the median score on the questionnaire and the age variable (Table 5).

The PACES scale dimensions and score do not seem to be related to age, as no significant differences were observed in Spearman’s Rho test. Regarding the reliability of the questionnaire when applied in the present study, the values obtained can be defined as satisfactory (Total = 0.89; Positive = 0.81; Negative = 0.83).

It should also be noted that the responses obtained were minimally distributed, with most scores showing a ceiling effect.

## 4. Discussion

The project was born from the need to know whether the sociodemographic characteristics of children can influence parents’ satisfaction with PA in the natural environment. The results showed significant differences in four items when comparing their scores among the three different age groups of the children. However, no statistically significant differences in item responses were observed according to age. In addition, dimension and total scale ratings do not appear to be influenced by age. Moreover, age showed no relationship with the dimensions and total score of the scales when evaluated. Finally, the ceiling effect observed in questionnaire responses has also been highlighted in previous studies, such as that of Kamnardsiri et al. [43], in assessing the enjoyment of an interactive game in older adults. Likewise, Ryuh et al. [44] observed this effect when promoting PA through exergaming in people with intellectual disabilities. On the other hand, Soylu et al. [45] did not find this ceiling effect when administering PACES after performing different aerobic training methods in adults.

The decision to become a parent has been recognized as a significant life event with considerable time demands that appear to have a significant impact on PA and the decision to leave sports, particularly for women [46]. In addition, the different perceptions that children have of natural areas can modify the frequency of visits and their parents’ opinion of them [47]. A relatively recent study indicates that having children at home is positively associated with a preference for PA in nature [5]. Similarly, many studies have shown associations between parental stress related to reports of child behavioral and emotional problems, adjustment difficulties, and internalizing or externalizing problems [48], which can be reduced by exposure to nature by both parties [10]. Sleddens et al. [49] pointed out that the relationship of influence on PA of parents and children is bidirectional, despite the fact that the vast majority of the literature only explores the influence of parents on their children. Yang and coworkers found that positive affection on the part of sons predicted greater PA practice on the part of their mothers [50]. Song et al. [51] also confirmed these findings, pointing out that the association is much greater when the child is between 3 and 6 years of age.

Regarding the positive items (6: “it gives me energy, 10: I get something extra, 14: It gives me strong feelings and 15: I feel good”), it can be observed that significant differences are found in the satisfaction that parents have with respect to PA in relation to their children’s age. It is well known that parents/carers want to pass on this tradition of PA to their daughters and sons, either due to the health benefits it may have or just for the satisfaction it brings to those who engage in PA [52]. Children can already create other worlds at the age of three [53] because of the development of their fine motor abilities, which enable them to manipulate their environment in a play area with sand, dirt, water, and loose pieces [54], which may explain the influence of age. As children are encouraged to explore and learn, chance also facilitates spontaneous exploration, which links physical and cognitive growth [55]. It should be mentioned that including children in activities has a favorable impact on parenting and other habits, such as engaging in fitness-related activities [56]. Moreover, a greater role of parents may be expected in preschool-aged children’s daily activity choices due to children’s early developmental stages [57]. However, parents of young children report less enjoyment with open areas than those without young children, according to a study [58].

There are no differences in gender, but in other studies [59], there is evidence that fathers have a greater influence on sons and mothers on daughters. According to Kirsten et al. [60], fathers were more inclined to utilize their personal conduct to encourage physical exercise in their children, whereas mothers affected their children’s PA by offering logistical support. This suggests that parental encouragement of their children’s PA affects both sexes differentially, which is consistent with Trost et al. [61]. On the other hand, the negative items do not seem to be influenced by the age of the children or by gender. However, if fathers are sedentary, they influence both genders, although to a greater extent in daughters, while mothers influence but not as much as fathers [62]. In general, there is no difference between parents’ satisfaction with PA and the gender of their children, as highlighted by another study, since this association is relative [57].

### Limitations and Future Lines of Research

As with other studies, this one has a number of limitations. Firstly, the sample selected was only of parents from the Community of Extremadura whose children were enrolled in the second cycle of Infant Education (3–5 years); therefore, there are sociocultural variables that may affect the responses obtained, such as the ceiling effect observed in the questionnaire scores. Second, because the sample was chosen at random, care should be taken while presenting the results. Finally, it is critical to draw attention to the dearth of prior research examining these issues. However, this study breaks with the previous trend of analysis by assessing how children affect parents’ satisfaction with PA rather than the other way around, so this research provides valuable preliminary information.

Some potential areas of research include expanding the sample to a national level in all educational stages in order to understand the satisfaction that parents have when they engage in PA with their children and checking whether gender and age have an influence. To do this, a deal may be made with more researchers from the many autonomous communities to gather all required data. Future research should consider parents’ geographic backgrounds (rural vs. urban), how much time they spend engaging in nature activities with their children, their motivations for engaging in PA, and the most frequent obstacles they face.

## 5. Conclusions

This study shows that parents’ satisfaction with PA in nature may vary depending on the age of their children, although this variable does not correlate with the dimensions and the total score of the questionnaire. On the other hand, the gender of the child does not seem to be a determining factor in parents’ satisfaction with PA.

It is also necessary to highlight that parents exercise less due to work and family responsibilities [26]; however, those who continue to practice this activity in their leisure time enjoy it more, and it reminds them of when they were infants or when they started doing sports before they had more work and/or family burdens [63]. It is important to transmit a healthy lifestyle throughout life since if parents have motivations and habits of doing PA in the natural environment, it is more likely that their children will also have them. It is also essential to involve all administrations and the media to achieve this, as more support and information are needed.

## Figures and Tables

**Table 1:** Parental characteristics (N = 280).

Variable	Categories	N	%
Gender	Male	101	36.1
	Female	179	63.9
Age	Between 20 and 29	13	4.6
	Between 30 and 39	163	58.2
	Between 40 and 49	102	36.4
	Over 50	2	0.8

Note: N: Number; %: percentage.

**Table 2:** Children’s characteristics (N = 280).

Variable	Categories	N	%
Gender	Male	145	51.8
	Female	135	48.2
Academic Year	First course	27	9.6
	Second course	111	39.6
	Third course	142	50.7
Age	3 years	27	9.6
	4 years	111	39.6
	5 years	142	50.7

Note: N: Number; %: percentage.

**Table 3:** Descriptive analysis and variations in the parent questionnaire items according to gender and age.

		Gender of Child			Age			
Item	Total	Female	Male		3 Years	4 Years	5 Years	
When I’m active…	M_e_ (IQR)	M_e_ (IQR)	M_e_ (IQR)	*p*	M_e_ (IQR)	M_e_ (IQR)	M_e_ (IQR)	*p*
1. I enjoy	5 (1)	5 (1)	5 (1)	0.255	5 (1)	5 (1)	5 (1)	0.623
2. I get bored	5 (0)	5 (0)	5 (0)	0.963	5 (1)	5 (0)	5 (0)	0.379
3. I don’t like it	5 (0)	5 (0)	5 (0)	0.744	5 (0)	5 (0)	5 (0)	0.322
4. I find it enjoyable	5 (1)	5 (1)	5 (1)	0.652	5 (1)	5 (1)	5 (1)	0.529
5. It’s no fun at all	5 (0)	5 (0)	5 (0)	0.614	5 (0)	5 (0)	5 (0)	0.093
6. It gives me energy	5 (1)	5 (2)	5 (1)	0.878	4 (2)	5 (1)	5 (1)	0.003
7. It depresses me	5 (0)	5 (0)	5 (0)	0.990	5 (0)	5 (0)	5 (0)	0.351
8. It is very pleasant	5 (1)	5 (0)	5 (1)	0.574	5 (1)	5 (0)	5 (1)	0.485
9. My body feels good	5 (0)	5 (0)	5 (0)	0.990	5 (0)	5 (0)	5 (0)	0.305
10. I get something extra	5 (1)	5 (1)	5 (1)	0.407	4 (0)	5 (1)	5 (1)	0.000
11. It is very exciting	4 (1)	4 (1)	4 (1)	0.652	4 (1)	4 (1)	4 (1)	0.822
12. It frustrates me	5 (0.75)	5 (1)	5 (0.50)	0.898	5 (1)	5 (0)	5 (0)	0.330
13. It’s not interesting at all	5 (0)	5 (0)	5 (0)	0.484	5 (0)	5 (0)	5 (0)	0.421
14. It gives me strong feelings	5 (1)	5 (1)	5 (1)	0.690	5 (0)	5 (1)	5 (1)	0.001
15. I feel good	5 (0)	5 (0)	5 (0)	0.933	5 (0)	5 (0)	5 (0)	0.047
16. I think I should be doing something else	5 (1)	5 (1)	5 (1)	0.215	4 (1)	5 (1)	5 (1)	0.272

Note: Me = median value; IQR = interquartile range. Each score obtained in the dimensions is based on a Likert scale (1–5).

**Table 4:** Differences in dimensions by gender and age.

	Gender				Age			
	Me (IQR)	Female	Male	*p*	3 Years	4 Years	5 Years	*p*
PACES Positive	4.7 (0.44)	4.7 (0.44)	4.7 (0.56)	0.677	4.6 (0.44)	4.7 (0.44)	4.7 (0.47)	0.673
	MeΣ (IQR)	Female	Male	*p*				
PACES Positive	42 (4)	42 (4)	42 (5)	0.677	41 (4)	42 (4)	42 (4.25)	0.673
	Me (IQR)	Female	Male	*p*				
PACES Negative	4.8 (0.43)	4.8 (0.43)	4.8 (0.43)	0.611	4.8 (0.29)	4.8 (0.43)	4.8 (0.43)	0.895
	MeΣ (IQR)	Female	Male	*p*				
PACES Negative	34 (3)	34 (3)	34 (3)	0.611	34 (2)	34 (3)	34 (3)	0.895
	Me (IQR)	Female	Male	*p*				
PACES Total	4.7 (0.38)	4.7 (0.38)	4.7 (0.38)	0.729	4.7 (0.38)	4.7 (0.38)	4.7 (0.38)	0.762
	MeΣ (IQR)	Female	Male	*p*				
PACES Total	76 (6)	76 (6)	76 (6)	0.729	75 (6)	75 (6)	76 (6)	0.762

Note: MeΣ = median of total questionnaire scores; Me = median value; IQR = interquartile range. Each score obtained in the dimensions is based on a Likert scale (1–5).

**Table 5:** Correlations between the age variable and PACES dimensions.

Dimensions	Age ρ (*p*)
PACES Positive	0.03 (0.577)
PACES Negative	0.02 (0.708)
PACES Total	0.04 (0.479)

Each score obtained in the dimensions is based on a Likert scale (1–5).

## Data Availability

The datasets are available through the corresponding author upon reasonable request.
